# The efficiency of self-regulation training program for coping with distant work stress under COVID-19 lockdown

**DOI:** 10.1192/j.eurpsy.2021.1057

**Published:** 2021-08-13

**Authors:** A. Kuznetsova

**Affiliations:** Faculty Of Psychology, Lomonosov Moscow state University, Moscow, Russian Federation

**Keywords:** stress-management, COVID-19, distant work stress, self-regulation

## Abstract

**Introduction:**

Under COVID-19 lockdown, mostly all organizations in non-productive sphere had to implement distant work forms. The personnel obligatory and rapidly switched to unknown work conditions and faced new stressors: COVID-19 fears, unstable internet connections, tensed communications, permanent noise, work hours extension. In order to cope with increased daily stress, the new version of self-regulation training program (Leonova, Kuznetsova, 2019) was implemented in distant format in order to train people: to evaluate the impact of distant work stressors; to measure stress manifestations during work hours; to choose self-regulation skills, effective for distant stress reduction.

**Objectives:**

In order to verify the distant training program, the empirical study was conducted, targeted to estimate effectiveness of self-regulation means during COVID-19 pandemic period.

**Methods:**

The program included progressive relaxation exercises as means for anxiety reduction and negative emotions control, and autogenic exercises for achievement an optimal for different work situations mental state. The empirical data were obtained by diagnostic methods for self-assessment of the main distant stress manifestations: anxiety and high fatigue (Spielberger, 1994, Leonova, 2012).

**Results:**

The program verification was conducted in employees of municipal administration offices (n = 214). The empirical data revealed high effectiveness of relaxation and autogenic means in decrease of anxiety (t=8,64; p<0,001) and fatigue (t=9,18; p<0,001).

**Conclusions:**

The first variant of distant program could be recommended for stress-management under pandemic lockdown. At the same time, advanced evaluating procedures are necessary to measure the coping effect of such programs, and to prove stress-reduction capacities of specialized distant training modules.

